# O085: A network-based approach using intra-hospital patient transfers to identify high-risk wards during nosocomial outbreaks

**DOI:** 10.1186/2047-2994-2-S1-O85

**Published:** 2013-06-20

**Authors:** M Ciccolini, J Arends, H Grundmann, AW Friedrich

**Affiliations:** 1 Medical Microbiology and Infection Control, University Medical Center Groningen, University of Groningen, Groningen, The Netherlands

## Introduction

Initial detection of a nosocomial outbreak can sometimes occur only after a considerable time has passed since the appearance of the index case(s). During this high-risk period from emergence to detection, within-hospital patient movements can disseminate the nosocomial pathogen to different admission wards. Following outbreak detection, a rapid, robust estimate of potentially exposed wards is of crucial importance in order to focus the implementation of infection prevention measures.

## Methods

We employ a mathematical approach, together with detailed patient location data and information on suspected cases, to estimate the potential number of exposed wards during an outbreak high-risk period. The model allows for different patient-to-patient transmission probability depending on time since last exposure, relative order in the transmission chain (first, or higher order contact), and on whether patients were located in the same room.

Model output consists of a risk score associated with each ward, and an exposure network, defined as all the exposed wards, together with precise information on dangerous contacts between them. Standard software was employed to visualize the exposure network growth throughout time.

## Results

This framework was successfully applied during a recent multi-resistant *K. pneumoniae* outbreak at a large university hospital in the Netherlands. A 4 month high-risk period resulted in 35 (out of 59) potentially exposed wards. The 10 wards with the highest model-calculated risk score were selected for post-exposure microbiological screening, which resulted in 154 additional screened patients. Further patients were screened, as controls, on other wards not included in the calculation. The complete exposure network was reconstructed, and the potential maximum reach of the outbreak was estimated. No additional positive patients were found and the outbreak was stopped.

## Conclusion

Due to the high level of patient exchange between different admission wards, determining their level of exposure during a prolonged high risk period rapidly becomes a complex task. Our network-based approach has been a valuable tool in reducing this complexity, focusing infection control interventions during an ongoing outbreak.

## Disclosure of interest

None declared.

